# Safe haven – a trauma-informed care model for oral health practitioners: a commentary

**DOI:** 10.1038/s41415-025-8862-5

**Published:** 2025-11-28

**Authors:** Kristy S. Choi, Woosung Sohn, Delyse Leadbeatter

**Affiliations:** School of Dentistry, Faculty of Medicine and Health, University of Sydney, Australia

## Abstract

There is a high prevalence of traumatic events in our society and the number of trauma sufferers in the general population is significant, with lasting adverse impacts on health, including oral health. Despite the high likelihood of oral health practitioners encountering patients with trauma, trauma history may not be disclosed or evident. In healthcare, trauma-informed care is a comprehensive and multi-level approach to patient care based on the understanding of the widespread nature and the impact of trauma. Trauma-informed care has not been widely implemented in oral health and multiple yet limited definitions of trauma could be further impeding implementation of trauma-informed care in oral health. To respond to these issues, we propose a definition of trauma in trauma-informed care which directly relates to the oral healthcare setting. We also propose a model – ‘Safe haven – a trauma-informed care model for oral health practitioners' – which aims to encourage oral health practitioners to practise trauma-informed care as a universal precaution for all patients in everyday practice.

## Key points

- Trauma-informed care in healthcare is a patient care approach to support patients with trauma.
- A definition of trauma in trauma-informed care for oral health is proposed as a patient's personal response to experiencing distressing life events which has lasting adverse effects on all aspects of a patient's life, including oral health and access to oral healthcare.
- ‘Safe haven – a trauma-informed care model for oral health practitioners' is presented, enabling oral health practitioners to provide oral healthcare which is sensitive to trauma.

## Introduction

Trauma is common.^1^ Studies have found that 70–90% of the general population have experienced at least one traumatic life event, such as war, disasters and violence,^2^^,^^3^ and individuals can respond to the traumatic experience as trauma. While traumatic events were once considered as an extreme abnormality outside of usual life experiences,^4^ relatively common distressing life events can also be traumatic, for example, illness, bullying, loss of a loved one, family dysfunction, and medical treatments,^5^ including distressing dental treatments and dental trauma. The high prevalence of traumatic events in our society means anyone could be suffering trauma knowingly or unknowingly and may be impacted by its effect.

Trauma can have a profound impact on oral health and patients with trauma can present with various challenges in oral healthcare. Patients commonly suffer orofacial injuries from domestic violence^6^ and child abuse^7^ and oral health practitioners may be the first point of care for the injuries. In addition to the physical injuries, the patients in this situation may also be suffering trauma from experiencing the traumatic event(s). Patients with trauma have the tendency to avoid preventive treatments, with frequent rescheduled, missed and cancelled appointments in the oral health setting.^8^^,^^9^^,^^10^ Lying down under an authoritative figure, the smell of the latex and many other common settings in oral health can trigger flashbacks of past traumatic events, such as sexual abuse.^10^^,^^11^ Receiving treatment from a practitioner wearing a white coat, the use of sharp and metallic instruments and bright head lights have also been shown to provoke trauma reactions for some patients with trauma history.^12^^,^^13^ In addition to many challenging situations, physical closeness between the oral health practitioner and the patient, which is unavoidable in oral healthcare, has the potential to make the patient feel vulnerable and unsafe, triggering past traumatic memories and potentially re-traumatising the patient in the oral health setting. Patients with trauma have shown dental fear and anxiety, increased gagging,^14^^,^^15^^,^^16^^,^^17^ high rates of various co-morbid mental illnesses, and significant disruption in body systems with chronic inflammation,^15^^,^^18^^,^^19^^,^^20^ which can all compromise provision of optimal oral health services. Common trauma-coping strategies, such as smoking, substance use, and poor diet can result in long-term negative oral health consequences, such as periodontitis, dental caries and oral infections.^8^^,^^21^^,^^22^^,^^23^ Difficulty to trust and self-regulate in patients with trauma can also adversely affect the therapeutic relationship between the oral health practitioner and the patient, which can compromise the quality of care and the treatment outcome.^24^

Treating patients with trauma can also place practitioners at risk of secondary traumatic distress where the practitioners are indirectly affected by the impact of trauma with similar symptoms as patients with trauma. Secondary traumatic distress can lead to chronic fatigue, disturbing thoughts, poor concentration, emotional detachment, exhaustion, avoidance and physical illnesses, and practitioners may struggle to provide high-quality care, even leading to mental health issues and burnout,^25^^,^^26^^,^^27^ having lasting adverse psychological impact.

Trauma-informed care is an evidence-based approach to patient care which incorporates understanding of trauma into all practices, thus minimising the risk of harm and re-traumatisation while providing care.^1^ Trauma-informed care can also improve the health and wellbeing of practitioners by reducing the risk of secondary traumatic distress. Studies in emergency medicine,^28^ paediatrics^29^ and nursing^11^^,^^30^ have all shown a positive impact of trauma-informed care on both practitioners and patients with increased quality of care while treating patients with trauma. Despite its profound importance, trauma-informed care has not been widely implemented in oral health.^31^ This paper aims to raise awareness among oral health practitioners to the universality of trauma and describe approaches to patient care which can promote the wellbeing of patients with trauma and of practitioners treating patients with trauma.

## Defining trauma for oral health

In oral health, the word ‘trauma' is commonly used for orofacial injuries. Among oral health practitioners, the term dental trauma is often used to describe the experience of past distressing dental treatments. In oral health, without broader understanding of trauma, trauma-informed care can be easily misinterpreted as treatment for orofacial injuries or caring for patients who have experience of past traumatic dental treatments. Trauma-informed care could also be considered as management of patients who have experienced specific traumatic events, such as domestic violence, or regarded as a mental health intervention for psychological trauma outside of the usual scope of oral health services. Such misunderstandings can diminish the awareness and implementation of trauma-informed care in oral health and lead to compromised care when treating patients with trauma.

Defining the word ‘trauma' could assist in better understanding trauma-informed care in oral health. Yet, there is no consensus on a common definition of trauma and its definition tends to be dependent upon the discipline which defines it. According to the American Psychiatric Association,^32^ trauma involves ‘actual or threatened death, serious injury, or sexual violence/violation'. In medicine, trauma can be defined as physical injuries,^33^ traumatic events/circumstances,^34^ or an individual's psychological response to events or injuries.^35^ In contemporary behavioural science, definition of trauma is limited as ‘an emotional response to a terrible event'.

According to the Substance Abuse and Mental Health Service Administration (SAMHSA),^1^ trauma can result from, ‘an event, series of events, or set of circumstances that is experienced by an individual as physically or emotionally harmful or life-threatening and that has lasting adverse effects on the individual's functioning and mental, physical, social, emotional, or spiritual wellbeing'.

While this most commonly accepted definition of trauma by SAMHSA is broader and more inclusive, it describes what trauma results from, not what trauma actually is. Such multiple and limited definitions of trauma can lead to confusion and compromise the understanding of trauma-informed care in oral health. To respond to the issue, we propose a definition of trauma in trauma-informed care for oral health which could, as a result, improve the understanding of trauma-informed care in oral health.

Table 1 defines ‘trauma' in trauma-informed care for oral health based on the three Es of trauma by SAMHSA,^1^ and the recognition that trauma is an integrated response of a whole person. For a clinical diagnosis of trauma, three Es of trauma are identified: traumatic Events, individual's Experience of the event as traumatic, and lasting adverse Effects of trauma. Trauma is a personalised experience of the event which is actual or threatened physical or psychological harm. Whether an individual interprets the experience as traumatic or not would depend on how the individual attributes meaning to, and how much they are physically and psychologically affected by, the event.^1^ How an individual experiences the event can also be influenced by a range of factors, such as cultural beliefs, life history, resilience, guilt, responsibility the individual imposes on the event, availability of social support, and the developmental stage.^1^^,^^36^^,^^37^ Trauma has lasting adverse impacts on all aspects of life, including mental, physical, social, emotional and spiritual wellbeing, and the effect can be immediate or delayed, with fear, anxiety, helplessness, horror, inability to cope with normal stress levels and difficulty in trusting others. Previously discussed oral health impacts of trauma are also summarised in Table 1.

**Table 1:** Definition of trauma in trauma-informed care for oral health. Trauma is a patient's personal response to experiencing distressing events which has lasting adverse effects on all aspects of their life, including oral health and access to oral healthcare

**Event**	**Experience**	**Effect**
Actual or threat of physical or psychological harm	Personal and subjective response which determines whether the event is traumatic or not Contributed by how the individual labels and assigns meaning to and is disrupted physically and psychologically by the event Influenced by a range of factors such as cultural beliefs, life history, resilience, guilt, responsibility the individual imposes on the event, availability of social support and the developmental stage	Lasting adverse effects on the individual's functioning and mental, physical, social, emotional, or spiritual wellbeing Can be an immediate or delayed onset, e.g., fear, anxiety, helplessness, horror, inability to cope with normal stresses and trust others
		Oral health impacts of trauma: Avoidance of preventive treatments and frequent reschedules, missed and cancelled appointments Various and common oral health settings can trigger traumatic memories and re-traumatise the patients Dental fear and anxiety Increased gagging Complex medical history, mental illnesses and chronic inflammation Trauma coping strategies such as smoking, substance use and poor diet can increase the risk of periodontal diseases, dental caries and oral infections Difficult to trust and self-regulate, compromising the therapeutic relationship, quality of care and treatment outcome Oral health practitioners treating patients with trauma history can suffer from secondary traumatic distress

The individual's experience of a traumatic event would be personal and internal.^38^ Characterising an abstract concept which cannot be measured or quantified presents inherent challenges and defining trauma is no exception. Its challenge stems from the subjective nature of human responses and diverse interpretation across individuals. With this in mind, a definition of trauma in trauma-informed care is proposed for oral health. For oral health, trauma in trauma-informed care is defined as a patient's personal response to experiencing distressing life events which has lasting adverse effects on all aspects of life. including oral health and access to oral healthcare.

Many oral health practitioners work in solo or small practice settings with the most direct patient interactions compared to other support staff in the practice. While establishing trauma-informed care requires a multi-level approach of an organisation, trauma-informed care practices by the oral health practitioners at the clinician level would be an effective way to support patients with trauma and initiate implementation of trauma-informed care in oral health.

**Fig. 1:**
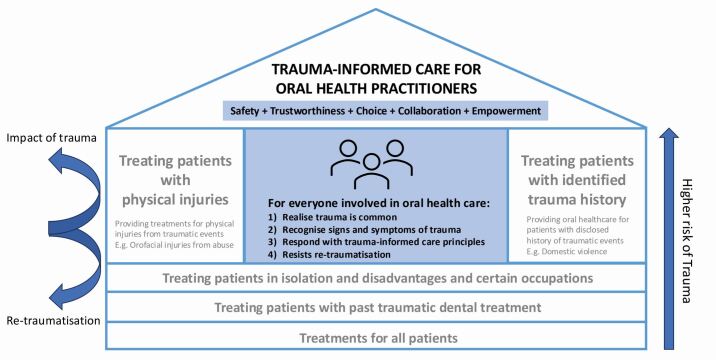
Safe haven – trauma-informed care for oral health

Figure 1 demonstrates an over-arching patient care approach to inform all interactions, decisions and treatments for oral health practitioners. The model features SAMHSA's four key competencies (the four Rs)^1^ of trauma-informed care: Realise the widespread impact of trauma; Recognise its signs and symptoms; Respond by trauma-informed care principles; and to Resist re-traumatisation. Trauma-informed care can protect everyone involved in the care from the adverse impacts of trauma and potential re-traumatisation.

Trauma-informed care practice by oral health practitioners begins by ‘Realising' that trauma is widespread and the impact of trauma is perverse. There is a high likelihood of oral health practitioners encountering patients with trauma and there is an increased awareness that everyone involved in the care may be affected by the impact of trauma, including the patient, their family members, the practitioner and other staff members, regardless of presence or absence of disclosure.^9^^,^^39^ Continuing education is an important and crucial element of trauma-informed care and common signs and symptoms of trauma should be learned and continually updated. However, challenging behaviours from patients with trauma may manifest in diverse and complex ways and it would be almost impossible to comprehend and interpret all the personal and individual signs and symptoms of trauma expressed by different patients. ‘Recognising' signs and symptoms of trauma in oral health means having an open and non-judgemental understanding that current behavioural challenges evident now may be a consequence of what has happened in the past. ‘Responding' means incorporating the principles of trauma-informed care – safety, trustworthiness, choice, collaboration and empowerment^40^ – into every aspect of care provided from the moment of first interaction. This means creating both a physically and psychologically safe and trusting environment by asking what can be done to make the patient more comfortable,^9^ informing the patient what will happen next,^39^ use of non-judgemental empathetic communication skills,^41^ asking for permission to touch or examine, and practising show-and-tell.^9^ Choice, collaboration and empowerment could be achieved by allowing additional appointment time to build rapport,^42^ or involving patients in the treatment planning process, where findings, options and recommendations are shared rather than imposed to provide a sense of control and ownership to build the patient's confidence.^43^ Addressing the practitioner's own history of trauma, discrimination and implicit biases are also very important in developing a non-judgemental outlook on patient behaviour and preventing re-traumatisation. ‘Resisting re-traumatisation' means for the practitioners to grow in awareness that any unexpected situations, for anyone in their care, can act as a trigger of past traumatic memories and has the potential to re-traumatise. Trauma-informed care requires active engagement by the oral health practitioner to resist harm by being committed to practising the principles of trauma-informed care throughout the entire encounter with the patient.

Infection control is practised in oral health for all patients as a universal precaution, regardless of the infection risk, history, or disclosure. This protects everyone involved in care from contagious and infectious diseases. Like infection control, trauma-informed care is recommended to be practised as a standard or universal precaution when treating all patients to create a sense of safety and to resist re-traumatisation. Just as there are patients with higher risk of infection and may require transmission-based precautions, patient populations with higher risk of trauma are also identified and more attention can be given to the need of trauma-informed care. While anyone is at risk of the exposure of traumatic events and trauma, patients with past traumatic dental treatments and patient populations suffering isolation and disadvantages, such as minority ethnic groups, women, children and young adults, refugees, First Nations people, and people in certain occupations, such as emergency service workers, are at higher risk of trauma, in ascending order, than the general population.^2^^,^^44^^,^^45^^,^^46^^,^^47^ Patients with physical injuries and disclosed trauma history have the highest risk of trauma. However, many patients do not feel the need to share their trauma history with oral health practitioners or are able to make connections between trauma and current challenges evident in oral healthcare.^19^ ‘Safe haven – a trauma-informed care model for oral health practitioners' acknowledges that trauma-informed care needs to be implemented for all patients, regardless of the risk, history or disclosure of trauma, to protect everyone from the impact of trauma, and aims to encourage oral health practitioners to practise trauma-informed care in everyday practice.

## Concluding remarks

According to Stalker *et al.*,^48^ patients with a trauma history are more likely to experience oral healthcare positively when practitioners have an understanding of trauma and how it can affect their interactions. With the high prevalence of traumatic events in our society, trauma-informed care should be practised by all oral health practitioners with the assumption of underlying trauma in everyone involved in oral healthcare. However, much of the current understanding of trauma-informed care is based on its principles and values rather than specific recommendations to guide clinical practice.^9^^,^^31^^,^^49^ The definition of trauma in trauma-informed care for oral health, and ‘Safe haven – a trauma-informed care model for oral health practitioners' in this commentary, aim to guide the oral health community towards a better understanding of trauma and trauma-informed care. Understanding trauma and implementations of trauma-informed care by oral health practitioners could avoid unnecessary stress and challenges for practitioners and prevent re-traumatisation of patients, enabling oral health practitioners to better respond to the needs of the individual patient and improve the general quality of care in oral health.
